# Genomic profiling of a DICER1-wildtype thyroblastoma reveals AGK-BRAF fusion, EIF1AX duplication, and TERT promoter mutations: integrated genomic and pathway analysis

**DOI:** 10.3389/fendo.2026.1747919

**Published:** 2026-04-22

**Authors:** Alberto Gualandi, Fernanda Picozzi, Annabella Di Mauro, Susi Pelotti, Imma D’Arbitrio, Pasquale De Luca, Lucia Cannella, Antonio Pizzolorusso, Alessandro Ottaiano, Annarita Peddio, Gerardo Ferrara, Salvatore Tafuto

**Affiliations:** 1 Pathology Unit, Istituto Nazionale Tumori IRCCS – Fondazione G. Pascale, Naples, Italy; 2 Sarcoma and Rare Tumors Unit, Istituto Nazionale Tumori IRCCS – Fondazione G. Pascale, Naples, Italy; 3 Unit of Legal Medicine, Department of Medical and Surgical Sciences, University of Bologna, Bologna, Italy; 4 Department of Science and Technology, Parthenope University of Naples, Naples, Italy; 5 Department of Clinical Medicine and Surgery, University Federico II, Naples, Italy

**Keywords:** AGK–BRAF fusion, DICER1-wildtype, EIF1AX, long-read sequencing, pathway enrichment, precision oncology, TERT promoter mutation, thyroblastoma

## Abstract

**Introduction:**

Thyroblastoma is a rare and highly aggressive embryonal thyroid malignancy typically associated with DICER1 alterations. However, DICER1-wildtype cases remain poorly characterized at the molecular level.

**Methods:**

We report a case of aggressive thyroblastoma in a 62-year-old male, negative for canonical DICER1 RNase IIIb mutations. Comprehensive genomic profiling was performed using Oxford Nanopore long-read sequencing, followed by integrative bioinformatic and pathway-level analyses.

**Results:**

Molecular analysis revealed an alternative oncogenic signature characterized by an EIF1AX p.Lys3_Lys5dup duplication, TERT alterations (promoter C228T and coding p.C42R), and an AGK–BRAF fusion predicted to drive constitutive MAPK/ERK signaling. Functional enrichment analyses highlighted dysregulation of translational initiation, telomere maintenance, and mitogenic pathways, alongside potential immune-escape mechanisms linked to DUX4 activation. Clinically, the tumor exhibited a triphasic morphology, extensive locoregional infiltration, pulmonary metastases, and only transient response to chemotherapy.

**Discussion:**

These findings expand the molecular spectrum of thyroblastoma beyond the canonical DICER1-driven paradigm and suggest that DICER1-wildtype cases may represent a distinct biological subgroup. The identification of alterations affecting TERT and MAPK pathways highlights potential therapeutic vulnerabilities and supports the clinical value of comprehensive genomic profiling in ultra-rare thyroid malignancies.

## Introduction

Thyroblastoma is an embryonal, high-grade thyroid neoplasm recently recognized as a distinct clinicopathological entity and characterized by primitive, multiphenotypic differentiation, aggressive local behavior, and a strong propensity for early dissemination (1–4). Histologically, thyroblastoma typically displays a heterogeneous architecture that may include epithelial, blastematous, and mesenchymal components, often recapitulating features of fetal thyroid development and ectomesenchymal differentiation (5–8). Clinically, patients frequently present with bulky cervical disease, airway or mediastinal involvement, and distant metastases at diagnosis, and the prognosis is generally poor despite multimodal management (2, 4, 9). Most reported cases harbor recurrent mutations in the *DICER1* gene, particularly affecting the RNase IIIb domain, and thyroblastoma has therefore been considered part of the expanding spectrum of *DICER1-*associated tumors (3, 4, 10–13). In this setting, *DICER1* alterations are thought to drive widespread microRNA dysregulation, developmental pathway reactivation, and cellular plasticity, thereby supporting the embryonal phenotype and rapid clinical progression (11, 14–16). DICER1 is not considered a driver gene solely because of its mutation frequency, but because multiple lines of genetic and functional evidence support a causal and selectively advantageous role in tumorigenesis. In DICER1-associated neoplasms, mutations show a highly recurrent and non-random pattern, typically involving hotspot missense variants in the RNase IIIb domain that impair 5p microRNA processing while preserving partial protein function. This stereotyped mutational profile, often occurring in a biallelic “two-hit” configuration (germline or somatic loss-of-function plus somatic hotspot mutation), indicates positive selection rather than passenger prevalence. Furthermore, functional studies demonstrate that RNase IIIb mutations lead to global microRNA dysregulation, derepression of oncogenic transcripts, and enhanced cellular proliferation, supporting a direct oncogenic mechanism (14). *DICER1*-wildtype thyroblastomas are exceptionally rare, and their genomic landscape remains essentially undefined (4, 12, 17). This represents a major diagnostic and biological gap, because the current diagnostic framework implicitly assumes *DICER1* alteration as a hallmark, and consequently, *DICER1*-intact tumors risk being misclassified as other high-grade thyroid or neck region malignancies (2, 13, 18). Parallel to this, advances in comprehensive molecular profiling — including long-read sequencing technologies, structural variant detection, and pathway-level functional annotation — are reshaping our understanding of rare aggressive tumors by uncovering gene fusions, promoter mutations, and non-canonical drivers that are often missed by routine hotspot panels (19–22). In particular, alterations in *EIF1AX*, TERT promoter activation, MAPK pathway lesions (including *BRAF* and *BRAF* fusion events), and deregulation of transcriptional repressors such as *DUX4* have emerged as potentially actionable or prognostically relevant signals in anaplastic, poorly differentiated, and other high-grade thyroid tumors (6, 15, 20, 21). Whether similar mechanisms can substitute for *DICER1* loss in thyroblastoma has not been systematically explored.

Here, we report an aggressive thyroblastoma in an adult patient, negative for canonical *DICER1* hotspot mutations on targeted analysis, and subsequently characterized through Oxford Nanopore long-read sequencing and integrative bioinformatic and pathway-level analysis. We describe the clinical course, radiologic features, histopathology and immunophenotype, and we define an alternative oncogenic program involving *EIF1AX* duplication, *TERT* promoter activation, *AGK–BRAF* fusion–mediated *MAPK/ERK* signaling, and *DUX4* dysregulation. We also discuss the diagnostic, biological, and therapeutic implications of these findings in the context of *DICER1*-wildtype thyroblastoma (2–4, 10, 12, 19–21).

## Materials and methods

### DNA extraction and sequencing

Genomic DNA was isolated from formalin-fixed, paraffin-embedded (FFPE) tumor tissue using the QIAamp DNA FFPE Tissue Kit (Qiagen, Hilden, Germany), a silica column–based extraction system optimized for fragmented or cross-linked nucleic acids. DNA concentration and purity were evaluated using a Qubit™ 4 Fluorometer (Thermo Fisher Scientific, USA) and the dsDNA HS Assay Kit, ensuring an A260/A280 ratio of 1.8–2.0 and a minimum yield of 10 ng/µL for downstream analysis. Library preparation was performed following the Oxford Nanopore Technologies (ONT) Ligation Sequencing Kit (Cat. No. SQK-LSK114, formerly 10170100) protocol, which includes DNA repair, end-preparation, and adapter ligation steps. Libraries were loaded onto R10.4.1 flow cells (Cat. No. FLO-MIN114, formerly 10170500) and sequenced on an ONT GridION platform, generating long-read 1D sequences in real time. Basecalling was conducted using Guppy (v6.5) in high-accuracy mode, and output FASTQ files were evaluated for read length distribution and quality metrics using NanoPlot (v1.41.3). Reads with a Phred quality score ≥10 were retained for downstream alignment and variant calling.

### Bioinformatic pipeline and variant analysis

Raw Oxford Nanopore sequencing data were basecalled using Guppy (v6.5) in high-accuracy mode to generate quality-filtered reads. Read quality metrics, including read length distribution and Phred score profiles, were assessed using NanoPlot. The resulting FASTQ files were aligned to the human reference genome (GRCh38/hg38) using Minimap2 (v2.26) with parameters optimized for long-read sequencing. Alignment statistics, including mapping rate and coverage distribution, were evaluated using SAMtools.

Small variant calling, including single-nucleotide variants (SNVs) and short insertions/deletions (indels), was performed using Medaka (v1.10), which generates consensus-based variant calls optimized for Oxford Nanopore long-read data. Structural variants (SVs >50 bp) were identified using Sniffles (v2.3) from the long-read alignments.

The initial variant call set was further refined using bcftools by applying quality-control filters based on read depth, mapping quality, and Phred-scaled variant quality score to retain high-confidence calls. Functional annotation of variants was performed using SnpEff (v5.2), which predicted the molecular consequences of each alteration (e.g., missense, nonsense, frameshift, splice-site, and regulatory variants).

Annotated variants were cross-referenced against curated genomic databases, including COSMIC for cancer-associated mutations, ClinVar for clinically interpreted variants, and dbSNP for known polymorphisms. Population allele frequencies from the gnomAD database were used to filter common germline polymorphisms, applying a minor allele frequency threshold of <0.001 in any population.

Because matched germline DNA was not available, variant interpretation relied on a tumor-only filtering strategy. Therefore, reported alterations are described as putative tumor-enriched variants rather than definitively confirmed somatic mutations. To minimize technical artifacts, only variants located on canonical chromosomes (chr1–22, chrX, chrY) were retained, and variants mapped to alternative contigs or low-confidence regions were excluded. Variants were further prioritized based on predicted functional impact, recurrence in cancer databases, and biological plausibility.

Read-level support for candidate variants was manually inspected using Integrative Genomics Viewer (IGV) to confirm consistent strand representation and exclude potential sequencing or alignment artifacts. Structural variant breakpoints and complex rearrangements, including the AGK–BRAF fusion, were additionally visualized using Ribbon. Downstream data processing, statistical summaries, and graphical visualizations were performed using Python (pandas, matplotlib, seaborn) and R (ggplot2).

A limitation of the present study is the absence of matched normal sequencing, which precludes definitive discrimination between somatic and rare germline variants. Nevertheless, the combination of population-frequency filtering, functional annotation, variant prioritization based on cancer-related databases, and manual read-level inspection supports the biological plausibility of the identified high-confidence candidate driver alterations, including the EIF1AX duplication, the TERT promoter hotspot mutation (C228T), and the AGK–BRAF fusion.

### Functional enrichment analysis

To investigate the biological significance of the identified genetic alterations, functional enrichment analysis was performed on the list of mutated genes. Analyses were conducted using g: Profiler (v0.3.5) with confirmatory evaluation in Enrichr, querying multiple repositories including KEGG, Reactome, and Gene Ontology (GO) categories (Biological Process, Molecular Function, and Cellular Component). Gene identifiers were standardized to official HGNC symbols prior to analysis. Statistical enrichment was evaluated using a hypergeometric test with Benjamini–Hochberg false discovery rate (FDR) correction for multiple testing. The background gene set consisted of all protein-coding genes annotated in the human reference genome (GRCh38). Pathways and GO terms with adjusted FDR < 0.05 were considered significantly enriched. Enrichment results were exported as tab-delimited files and visualized using R (v4.3.1) with the ggplot2 package. Only genes harboring high-confidence coding or splice-site variants were included in the enrichment analysis.

### Statistical analysis

Descriptive statistical analyses were performed to summarize sequencing metrics and variant characteristics. Continuous variables, including variant allele frequency (VAF), read depth, and variant quality scores, were summarized using median and range values. Given the single-case design of the study, no inferential statistical comparisons between groups were performed.

## Results

### Clinical presentation and radiology

**Figure 1:**
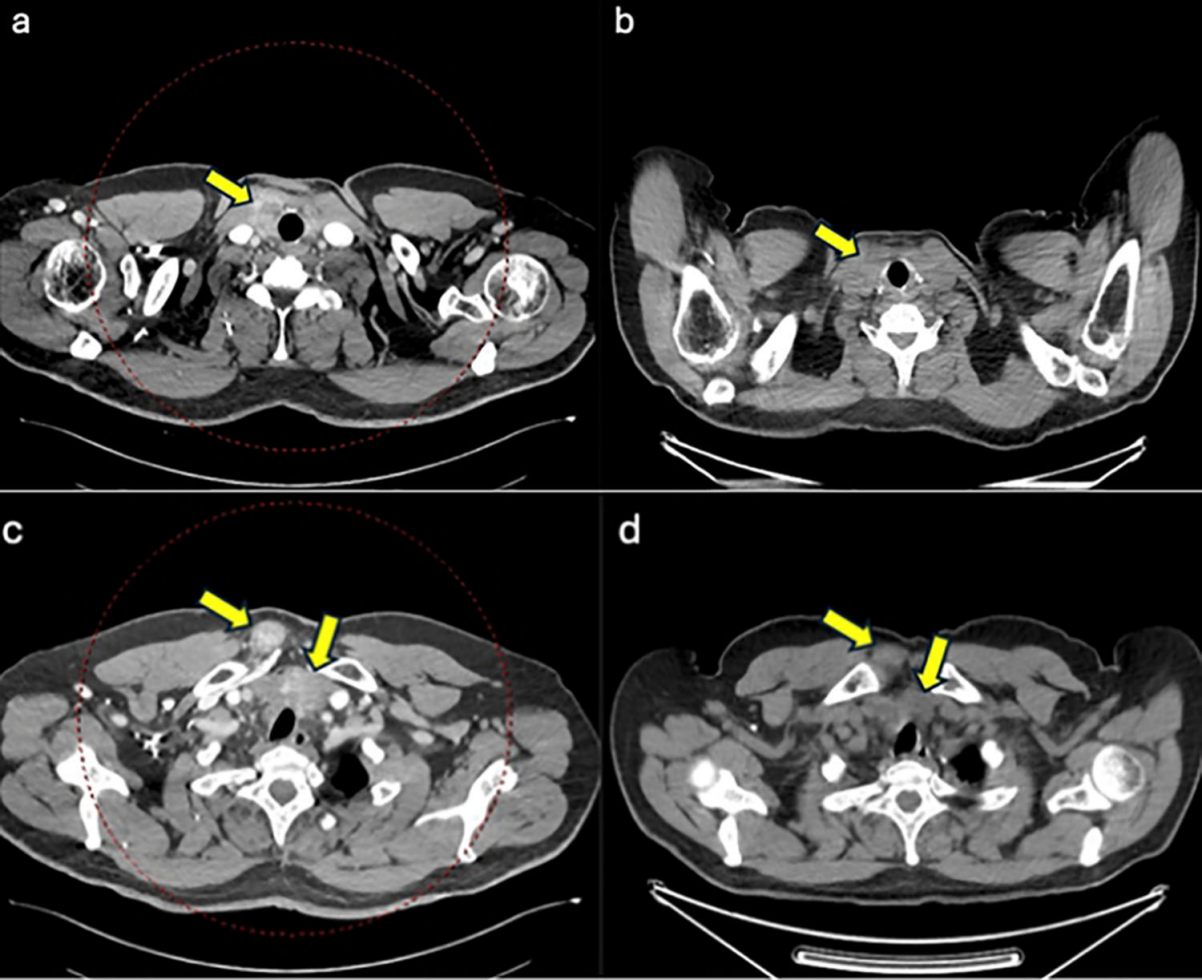
Representative pre- and post-treatment CT scans. **(a)** Contrast-enhanced CT (pre-treatment): The yellow arrow highlights heterogeneously enhancing pathological tissue in the right thyroid bed, in a patient with prior total thyroidectomy. The lesion (approximately 32 × 26 mm) infiltrates the prethyroid muscles and extends caudally into the anterior mediastinum, reaching a maximum size of 42 × 41 mm. **(b)** Post-treatment CT (non-contrast): The yellow arrow demonstrates a marked reduction in size and metabolic activity of the lesion, indicating a positive therapeutic response. No contrast-enhanced CT image was available at this time point because only PET/CT without intravenous contrast was performed for metabolic response assessment. **(c)** Contrast-enhanced CT (pre-treatment): The yellow arrows show additional pathological tissue in the presternal region (approximately 43 × 32 mm), infiltrating the right sternocleidomastoid muscle, which appears swollen compared to the opposite side. There is also posterior infiltration of the right half of the sternal manubrium, with focal cortical erosion. **(d)** Post-treatment CT (non-contrast): The yellow arrows indicate a significant reduction in both volume and metabolic activity of the pathological tissue, consistent with a good treatment response. Contrast-enhanced imaging was not available in the post-treatment setting, as PET/CT without intravenous contrast was used to monitor the metabolic outcome.

A 62-year-old man with a history of multinodular goiter underwent a total thyroidectomy due to a rapid increase in thyroid size, leading to recent onset of dyspnea and dysphagia. The histological findings were consistent with the diagnosis of thyroblastoma. A postoperative CT scan showed the presence of a cervical mass extending into the anterior mediastinum, infiltrating the right side of the sternal manubrium, right sternocleidomastoid and pectoralis muscles, with multiple metastatic nodes in the Barety lodge and in the aortopulmonary window infiltrating the trachea. Multiple pulmonary metastases were also found. The patient was referred to our center for pathological and molecular consultation. Postoperatively, chemotherapy with Epirubicin and Ifosfamide was initiated; after three cycles, a partial therapeutic response was achieved with dimensional reduction of the lesions. Representative CT scan and PET/CT images (**Figures 1a–d**) demonstrate the extent of local infiltration and the response after chemotherapy. Treatment was then discontinued due to cardiotoxicity, and the patient was subsequently managed with supportive care only, dying about one month later.

### Histopathology and immunophenotype

Macroscopic examination revealed that the right lobe was completely occupied by an 11 cm, gray-yellowish nodular lesion with cystic areas and indistinct margins. A second, smaller nodule measuring 0.7 cm was also identified in the lower third of the left lobe.

**Figure 2:**
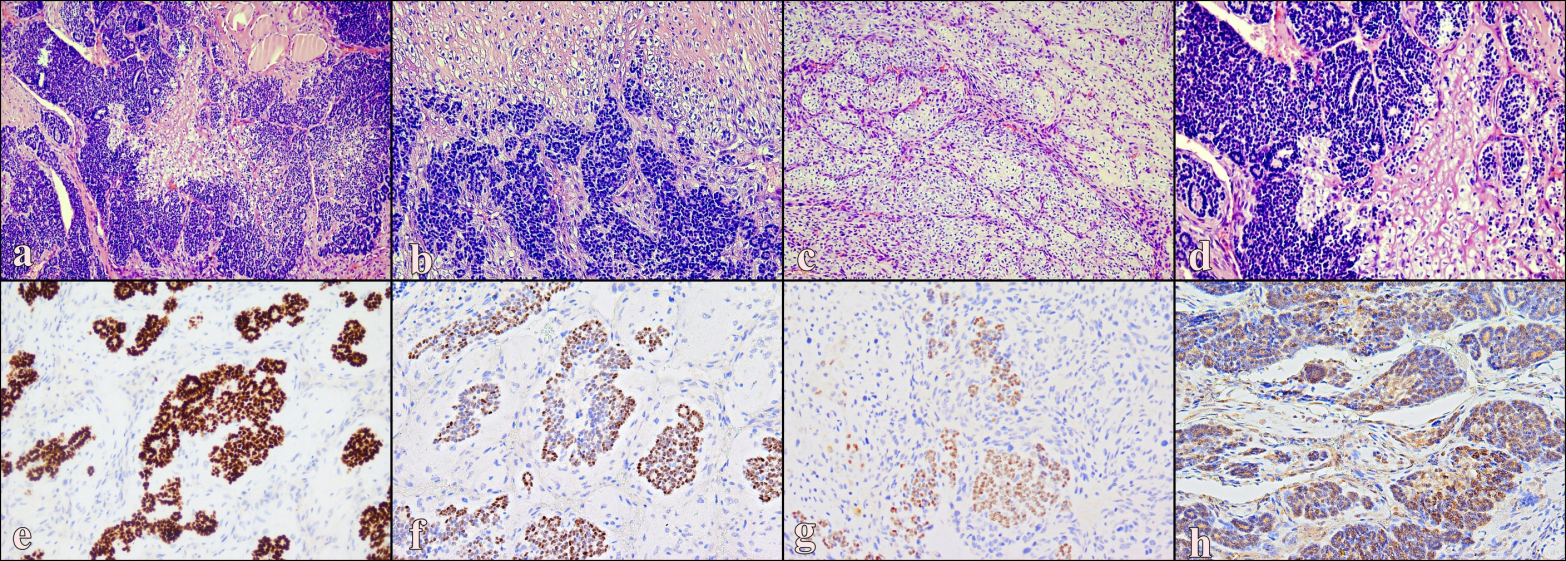
Representative histological and immunohistochemical features of thyroblastoma: **(a)** Low-power view showing an infiltrative triphasic neoplasm composed of follicular, blastematous, and mesenchymal components (H&E, 10x). **(b)** Primitive follicular structures admixed with solid blastematous cells and atypical mesenchymal stroma (H&E, 20x). **(c)** Area of undifferentiated mesenchymal stroma with osteo-chondroid differentiation (H&E, 20x). **(d)** Embryonal follicles intermingled with blastema-like cellular aggregates and mesenchymal elements (H&E). **(e)** Strong nuclear TTF1 expression highlighting the embryonal follicular component (IHC, 20×). **(f)** Nuclear PAX8 positivity in the epithelial component, supporting thyroid lineage differentiation (IHC, 20×). **(g)** Cytoplasmic expression of CK AE1/AE3 in epithelial tumor cells (IHC, 20×). **(h)** Weak-to-moderate NSE expression in the blastematous and microfollicular components (IHC, 20×).

Histologically, the neoplasm displayed a heterogeneous, multifocal morphology with three intermixed components. The first component (**Figures 2a, b**) consisted of primitive microfollicular structures, lacking orderly lobular organization, with irregular connective septa and a rich vascular network. These structures were composed of small cells with hyperchromatic nuclei, containing small lumens with scarce, heterogeneous colloid. In some areas, they were arranged in compact clusters with poorly recognizable luminal spaces. Due to its characteristics, this neoplastic component resembled fetal thyroid tissue.

The second component (**Figure 2c**) was a solid area of primitive blastema composed of small round cells with scant cytoplasm. These blastematous cells were positive for TTF1, PAX8 (weak), and NSE (weak), but negative for Synaptophysin, SALL4, PLAP, Glypican3, OCT3/4, Inhibin A, and Calretinin.

The third component (**Figure 2d**) was mesenchymal, consisting of spindle and pleomorphic epithelioid cells, with partial differentiation toward immature osseous and chondroid lineages. These elements were positive for SATB2, CD31, and ERG.

Immunophenotypically, the primitive follicular component showed strong nuclear TTF1 expression (**Figure 2e**), PAX8 positivity (**Figure 2f**), and CK AE1/AE3 expression in epithelial cells (**Figure 2g**). NSE showed weak-to-moderate positivity in the blastematous and microfollicular components (**Figure 2h**). The tumor exhibited high mitotic activity (22 mitoses/mm²), scattered necrosis, and lymphovascular invasion. All three components were immunohistochemically negative for Calcitonin, S100, p40, CD117, D2-40, cMyc, SSX, and SS18-SSX.

### Genetic analysis

Whole-genome sequencing performed on tumor DNA revealed a complex and heterogeneous mutational profile. A total of 2,168 putative somatic variants were initially detected before filtering. Variant allele frequency (VAF) values ranged from 0.01 to 0.98, with a median VAF of approximately 0.46. The distribution displayed a trimodal pattern with peaks corresponding to low-frequency subclonal variants (<0.1), heterozygous events (~0.5), and high-frequency variants approaching clonality (>0.9) (**Supplementary Figure 1a**). Read depth was predominantly skewed toward low coverage, though a subset of variants reached depths above 1000 reads (**Supplementary Figure 1b**), potentially indicating focal amplifications or repetitive regions. Similarly, quality scores clustered below 5000, with a small fraction of high-confidence variants exceeding this threshold (**Supplementary Figure 1c**). The majority of detected variants mapped to autosomal chromosomes, with a notable enrichment on chromosomes 4, 13, and 16. Genomic variants were also unevenly distributed along chromosome coordinates, further reflecting intratumoral genomic instability (**Supplementary Figure 1d**).

**Figure 3:**
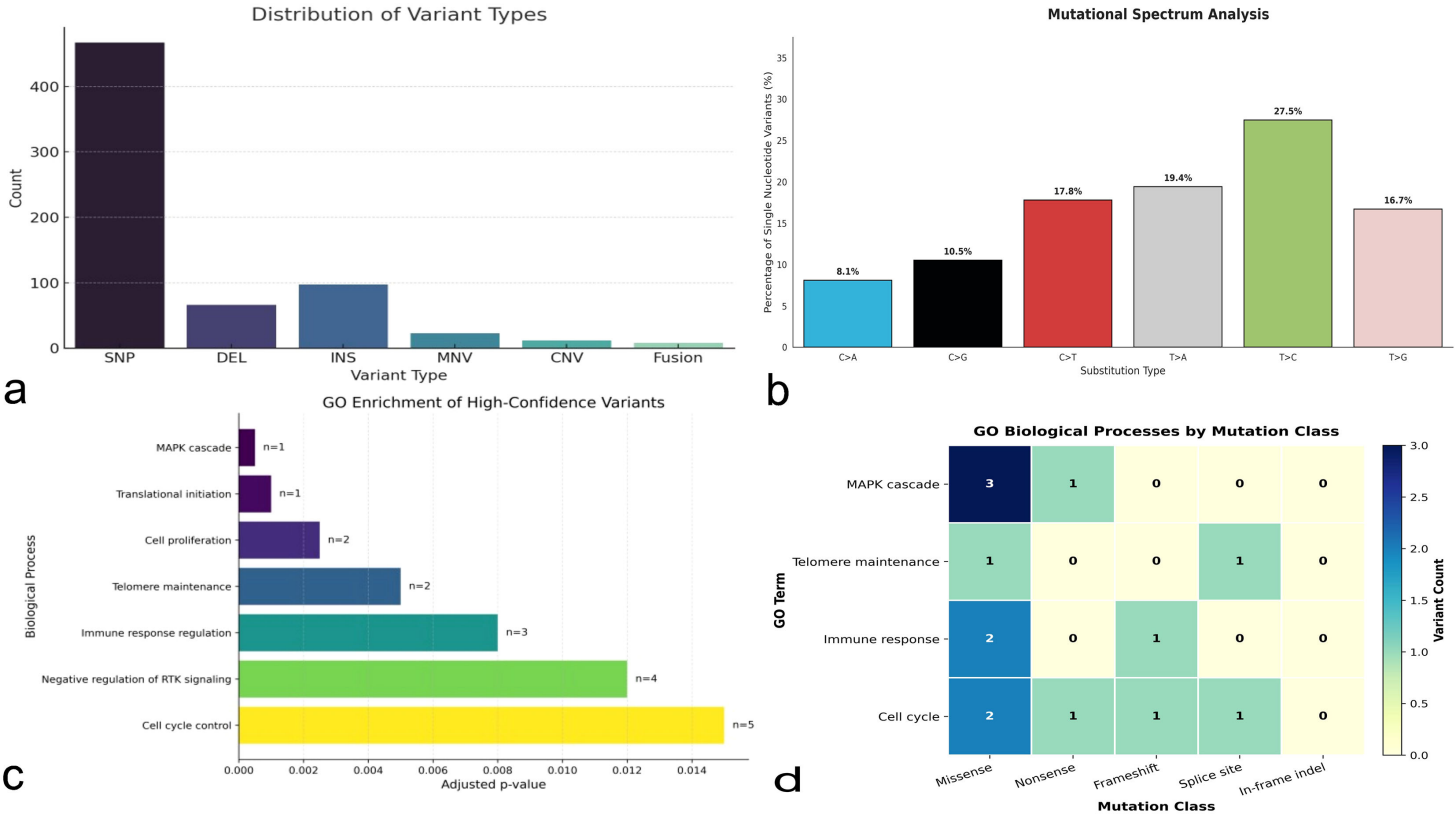
Overview of somatic variant types and their associated biological processes. **(a)** Distribution of variant classes, **(b)** Relative frequency of single nucleotide substitution types, showing the percentage contribution of each base substitution class (C>A, C>G, C>T, T>A, T>C, and T>G). **(c)** Gene Ontology (GO) enrichment analysis of genes harboring high-confidence variants. The horizontal bar plot reports enriched biological processes ranked by adjusted p-value (Benjamini–Hochberg correction). The number of genes contributing to each enrichment term is indicated next to each bar (n). **(d)** Heatmap showing the distribution of mutation classes across selected enriched biological processes. Values represent the number of variants associated with each mutation class within the corresponding GO category.

Variant types were mainly single nucleotide polymorphisms (approximately 47%), followed by insertions (~10%) and deletions (~7%) (**Figures 3a, b**). Following annotation and filtering, high-confidence coding and splice-site variants were selected using SnpEff, and cross-referenced with the COSMIC Cancer Gene Census and ClinVar databases. Among these, pathogenic or likely pathogenic variants were identified in both canonical cancer drivers and poorly characterized genes. Specifically, mutations were found in *EIF1AX* (p. Lys3_Lys5dup), *TERT* promoter (C228T), *TERT* (p.C42R), and a *BRAF* rearrangement (AGK-BRAF fusion). No pathogenic mutations were detected in the hotspot regions of the *DICER1* gene (exons 23–25). Additional variants were observed in less well-characterized loci such as *DUX4*, *OPCML*, *FAM230C*, and *ROCK1P1*. These alterations indicate the presence of both canonical oncogenic drivers and additional genomic variants.

### Functional annotation and GO analysis

The genes harboring pathogenic or likely pathogenic variants were subjected to Gene Ontology (GO) enrichment analysis. Significant enrichment was observed for terms related to translational initiation (linked to *EIF1AX*), telomere maintenance and cellular senescence (*TERT*), and MAPK signaling cascade regulation (*BRAF*) (**Figure 3c**). Additionally, *DUX4* was associated with transcriptional regulation of early embryonic genes and immune-related pathways, supporting a potential role in immune evasion mechanisms. *OPCML*, a putative tumor suppressor gene, was linked to negative regulation of receptor tyrosine kinase signaling, suggesting a modulating role in oncogenic signaling networks (**Figure 3d**).

### Functional and pathway analysis

An integrated functional and pathway analysis was performed, combining high-confidence variants derived from next-generation sequencing data with externally validated pathogenic alterations. The curated gene list included both rare or poorly characterized genes (e.g., *OPCML, DUX4, FAM230C, ROCK1P1, AC008103.3*) and clinically relevant drivers (*EIF1AX, TERT, BRAF*). Gene Ontology (GO) annotation revealed involvement in key biological processes such as translational initiation (*EIF1AX*), telomere maintenance and cellular senescence regulation (TERT), and signal transduction via the MAPK/ERK cascade (BRAF). Additionally, *OPCML* was associated with the negative regulation of receptor tyrosine kinases, while *DUX4* emerged as a transcription factor capable of activating early embryonic genes and modulating immune-related responses. Pathway enrichment analysis through databases such as KEGG, Reactome, and g: Profiler highlighted several key pathways including protein synthesis regulation, cell cycle control, and telomere elongation. Notably, *BRAF* was central to the RAS-RAF-MEK-ERK signaling cascade, a canonical pathway involved in cell proliferation and differentiation. The presence of *DUX4* suggested the activation of germline and immunomodulatory pathways, consistent with mechanisms of immune escape reported in certain rare tumors. This multi-level enrichment supports the functional relevance of both common and rare genomic alterations in shaping the tumor’s biology. The curated high-confidence variants and their clinical relevance are summarized in **Supplementary Table 2**.

### Clinical impact of molecular alterations

From a clinical and molecular standpoint, the *EIF1AX* duplication, detected at high variant allele frequency, is consistent with an early clonal event potentially affecting translational regulation. The coexistence of the *TERT* promoter mutation (C228T) (**Supplementary Figure 2a**) and the coding variant (C42R) suggests telomerase reactivation, a molecular feature frequently associated with biologically aggressive or poorly differentiated thyroid neoplasms.

**Figure 4:**
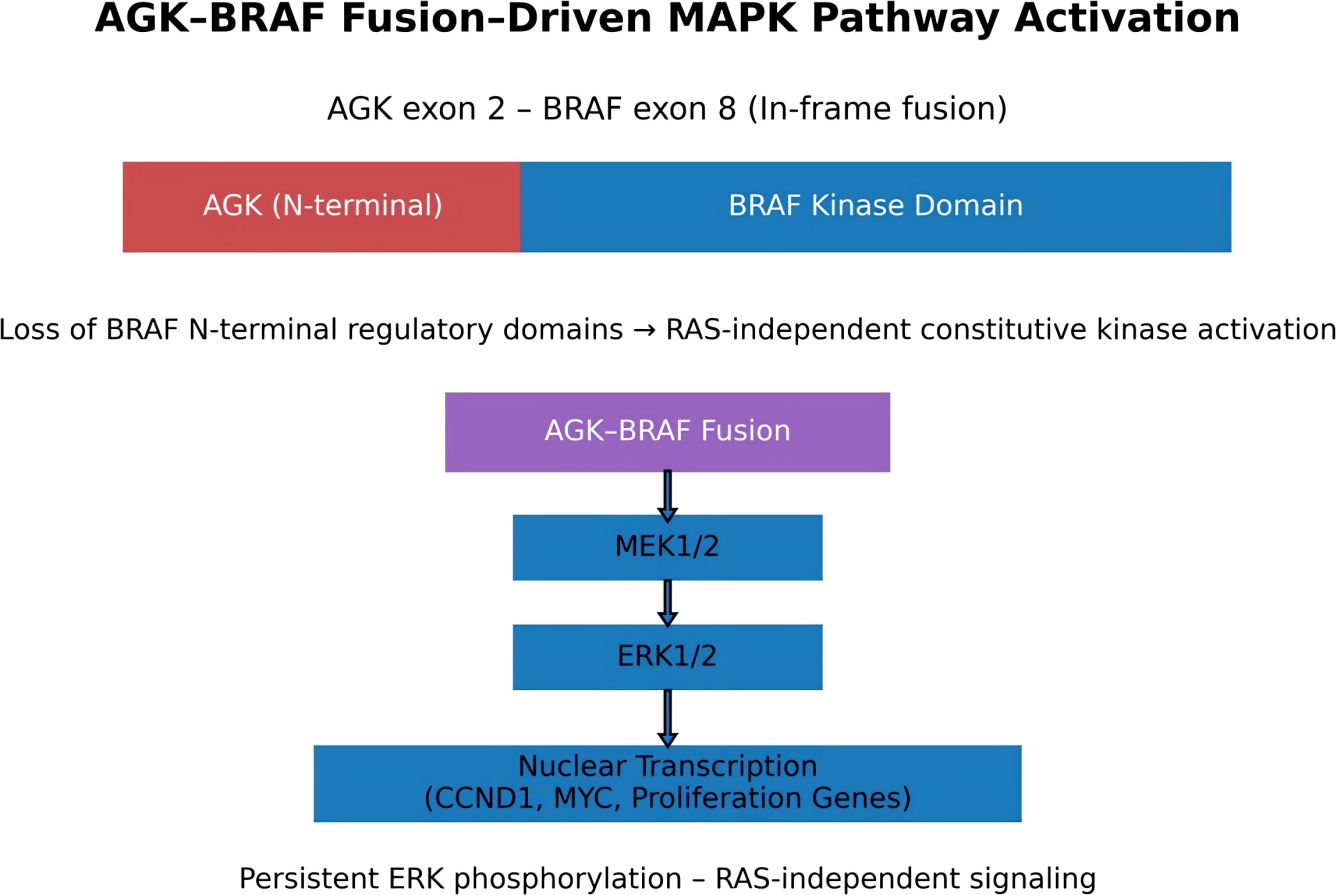
Structural and functional model of the AGK–BRAF fusion identified in this case.The rearrangement joins *AGK* exon 2 with *BRAF* exon 8, generating an in-frame fusion protein that preserves the catalytic serine/threonine kinase domain of BRAF while removing the N-terminal regulatory region responsible for RAS-dependent autoinhibition. This structural configuration results in constitutive, RAS-independent kinase activity. The fusion protein activates the MAPK signaling cascade through phosphorylation of MEK1/2 and ERK1/2, promoting transcription of genes involved in proliferation, survival, and tumor progression.

The *AGK–BRAF* rearrangement was supported by multiple split-read alignments spanning the breakpoint region in IGV. A schematic representation of the AGK–BRAF fusion and its downstream MAPK/ERK signaling cascade is provided in **Figure 4**. Consistent with this observation, the genomic distribution of breakpoint-supporting split reads across the *BRAF* locus on chromosome 7 demonstrated clustering of split-read signals compatible with the *AGK–BRAF* structural rearrangement identified by long-read sequencing (**Supplementary Figure 2b**). *DUX4*, a transcription factor normally epigenetically silenced in adult tissues, was also identified, further expanding the molecular heterogeneity of this case. In order to explore potential immune-related implications suggested by this finding, additional immunohistochemical analyses were performed. β2-microglobulin showed strong and diffuse membranous expression, whereas HLA class I demonstrated weak and focal staining in tumor cells. CD8 immunostaining revealed a limited and spatially heterogeneous intratumoral cytotoxic T-cell infiltrate, and PD-L1 expression was strong but focal (**Supplementary Figure 3**). Although these observations do not provide functional evidence of immune escape, they highlight a complex interplay between oncogenic signaling and immune-related pathways, reinforcing the translational relevance of comprehensive molecular profiling in rare thyroid malignancies.

Finally, the presence of alterations in *FAM230C* and *ROCK1P1*, whose biological significance remains to be clarified, underscores the heterogeneous and partially atypical genomic landscape characterizing DICER1-wildtype thyroblastoma.

Collectively, these findings illustrate the coexistence of established oncogenic drivers and less-characterized genomic events, reinforcing the molecular complexity of this rare entity.

## Discussion

Thyroblastoma represents a recently defined, high-grade embryonal thyroid malignancy characterized by multiphenotypic differentiation and aggressive clinical behavior. Histologically, it typically displays a heterogeneous architecture including epithelial (immature follicular), blastematous, and mesenchymal components, often recapitulating elements of early thyroid development (2–4). Historically, these tumors were frequently classified under different designations, including malignant thyroid teratoma or carcinosarcoma, before recognition of their distinctive clinicopathological and molecular features (3, 4).

A defining molecular hallmark of thyroblastoma has been the presence of recurrent mutations in the *DICER1* gene, particularly involving hotspot missense variants affecting the RNase IIIb domain (5–7). These mutations disrupt microRNA processing and lead to widespread dysregulation of gene expression programs, contributing to tumorigenesis through altered developmental signaling pathways (14–16). Consequently, thyroblastoma has traditionally been considered part of the broader spectrum of *DICER1*-associated tumors. However, a subset of cases lacks detectable *DICER1* mutations, highlighting the existence of alternative oncogenic mechanisms in this rare entity (3, 6, 17).

Recent studies have begun to characterize the molecular landscape of *DICER1*-wildtype thyroblastomas. Xu et al. reported that these tumors frequently lack canonical RNase IIIb mutations and instead harbor alternative oncogenic drivers, including alterations affecting MAPK signaling and telomere-related pathways (17). Similarly, Kim et al. demonstrated that aggressive thyroid tumors without *DICER1* mutations may rely on canonical oncogenic cascades rather than microRNA-processing defects (18). This molecular configuration contrasts with classical *DICER1*-mutant thyroblastomas, where impaired miRNA biogenesis represents the central pathogenetic mechanism (14–16). Importantly, it also differs from poorly differentiated thyroid carcinoma, which is typically characterized by mutations in *RAS*, *BRAF* V600E, *TP53*, and frequent *TERT* promoter alterations arising in a stepwise dedifferentiation model (23).

In the present study, long-read genomic sequencing revealed a constellation of alterations involving *EIF1AX*, *TERT*, and an AGK–*BRAF* fusion, defining a potential alternative oncogenic framework. The identification of these alterations expands the molecular spectrum of thyroblastoma and supports the concept that *DICER1*-wildtype cases may represent a biologically distinct subgroup.

Alterations affecting *TERT* further reinforce the aggressive biological phenotype observed in this tumor. The promoter mutation C228T generates *de novo* binding sites for ETS transcription factors and is strongly associated with poor prognosis in thyroid malignancies (35, 36). Because ETS transcription factors are responsive to MAPK signaling, constitutive ERK activation induced by the AGK–*BRAF* fusion may further amplify *TERT* transcriptional activity, promoting telomerase activation and replicative immortality (25–29, 35–37). The coexistence of promoter and coding *TERT* variants observed in our case suggests a convergent mechanism reinforcing telomere maintenance and cellular immortalization.

The *AGK–BRAF* fusion represents another key driver identified in this tumor. BRAF fusions are well-recognized oncogenic events capable of activating the MAPK/ERK signaling cascade independently of upstream RAS signaling (25–27). Although most clinical experience with MAPK-targeted therapy in thyroid cancer has focused on the *BRAF* V600E mutation, accumulating evidence indicates that *BRAF* fusion events can similarly drive constitutive pathway activation and may exhibit sensitivity to MEK inhibitors or combined MAPK-targeted therapies (25–29). The *AGK–BRAF* rearrangement has been reported in several tumor types, including pediatric papillary thyroid carcinoma and lung adenocarcinoma, and has been associated with enhanced proliferative signaling and tumor progression (28, 29). The presence of this alteration in thyroblastoma therefore raises the possibility of therapeutically actionable MAPK pathway activation.

To further contextualize this finding, *AGK–BRAF* fusions have been described as recurrent oncogenic events in pediatric thyroid carcinoma, where they promote constitutive MAPK activation and are associated with distinct biological behavior (38). More broadly, fusion-driven MAPK signaling represents a characteristic feature of pediatric and rare thyroid tumors, often differing from classical *BRAF* V600E-driven carcinomas in both molecular profile and clinical course (39). In this context, the identification of an *AGK–BRAF* fusion in thyroblastoma supports the hypothesis that alternative MAPK-activating mechanisms may operate in DICER1-wildtype embryonal thyroid tumors. Notably, according to the WHO Classification of Endocrine and Neuroendocrine Tumours (5th edition), thyroblastoma is currently the only recognized embryonal tumor of the thyroid, underscoring both its biological uniqueness and the lack of representation in large-scale genomic datasets.

The *EIF1AX* duplication identified in this case likely represents an early clonal event. *EIF1AX* encodes eIF1A, a translation initiation factor responsible for start-codon recognition and fidelity of the pre-initiation complex. Mutations in *EIF1AX* have been reported in advanced thyroid carcinomas, where they frequently co-occur with additional oncogenic alterations and contribute to tumor dedifferentiation and aggressive behavior (30–32). Mechanistic studies suggest that mutant *EIF1AX* enhances translation initiation and activates adaptive stress-response pathways involving *ATF4*, mTORC1 signaling, and *c-MYC* stabilization, thereby coupling translational control to metabolic reprogramming and proliferation (33, 34). The p. Lys3_Lys5dup variant affects the N-terminal region implicated in scanning dynamics and start-codon selection, potentially altering translational output and contributing to oncogenic signaling.

Functional enrichment analyses further supported the biological relevance of the detected alterations. Gene Ontology and pathway-level analyses highlighted dysregulation of translational initiation, telomere maintenance, and MAPK signaling pathways, consistent with the functional roles of *EIF1AX*, *TERT*, and *BRAF* (19–21, 25–37). Additional genes identified in the mutational landscape, including *OPCML* and *DUX4*, were associated with modulation of receptor tyrosine kinase signaling and transcriptional programs linked to developmental and immune-related pathways.

The detection of *DUX4* is particularly intriguing from an immunological perspective. *DUX4* is a transcription factor normally epigenetically silenced in adult somatic tissues but aberrantly expressed in certain malignancies (39–41). Experimental studies have shown that *DUX4* activation can suppress interferon-γ–responsive gene programs and impair antigen presentation through downregulation of MHC class I molecules, thereby promoting immune evasion (40–42). In our case, immunohistochemical analysis revealed preserved β2-microglobulin expression, partial reduction of HLA class I, limited CD8-positive T-cell infiltration, and focal PD-L1 expression. Although these findings do not provide definitive functional evidence of immune escape, they are consistent with previously described DUX4-associated immune-modulatory mechanisms. Given the single-case design and the absence of transcriptomic validation, these observations should be interpreted as hypothesis-generating and warrant further investigation through integrated immune profiling approaches.

Taken together, the coexistence of oncogenic alterations affecting translational control (*EIF1AX*), telomere maintenance (*TERT*), and MAPK signaling (*AGK–BRAF*), together with potential immune-modulatory mechanisms associated with *DUX4*, suggests a composite molecular architecture combining proliferative and immune-related signaling pathways. Although these observations derive from a single case, they may provide a biologically plausible explanation for the aggressive phenotype observed in this tumor and warrant further investigation in additional cases of *DICER1*-wildtype thyroblastoma.

Overall, our findings demonstrate that thyroblastoma can harbor biologically significant genomic alterations even in the absence of canonical *DICER1* mutations. The identification of an alternative molecular framework involving *EIF1AX*, *TERT*, and *AGK–BRAF* expands the current understanding of thyroblastoma pathogenesis and underscores the importance of comprehensive genomic profiling in the diagnostic evaluation of rare thyroid malignancies (19–21). Future studies involving larger cohorts will be necessary to determine the prevalence and clinical significance of these alterations and to clarify whether *DICER1-*wildtype thyroblastoma represents a distinct molecular subtype with specific therapeutic vulnerabilities.

## Conclusion

Taken together, this report describes an exceptional case of *DICER1*-wildtype thyroblastoma, a tumor type in which *DICER1* alterations are usually considered defining events. The coexistence of *EIF1AX, TERT*, and *AGK–BRAF* alterations supports alternative oncogenic mechanisms distinct from canonical *DICER1*-driven pathways. Functional analysis revealed dysregulation of translation, telomere maintenance, and MAPK signaling, defining a molecularly distinct subset. These findings highlight the importance of integrated histopathological and genomic profiling in clarifying diagnosis and revealing potential therapeutic vulnerabilities in ultra-rare thyroid malignancies.

## Data Availability

The datasets presented in this study can be found in online repositories. The names of the repository/repositories and accession number(s) can be found below: https://zenodo.org/records/17627658,17627658.
