# Supplementary figures and images for: Genomic profiling of a DICER1-wildtype thyroblastoma reveals AGK-BRAF fusion, EIF1AX duplication, and TERT promoter mutations: integrated genomic and pathway analysis
Source: Front Endocrinol (Lausanne). 2026 Apr 22;17:1747919. doi: 10.3389/fendo.2026.1747919 (PMC13143743; doi:10.3389/fendo.2026.1747919)

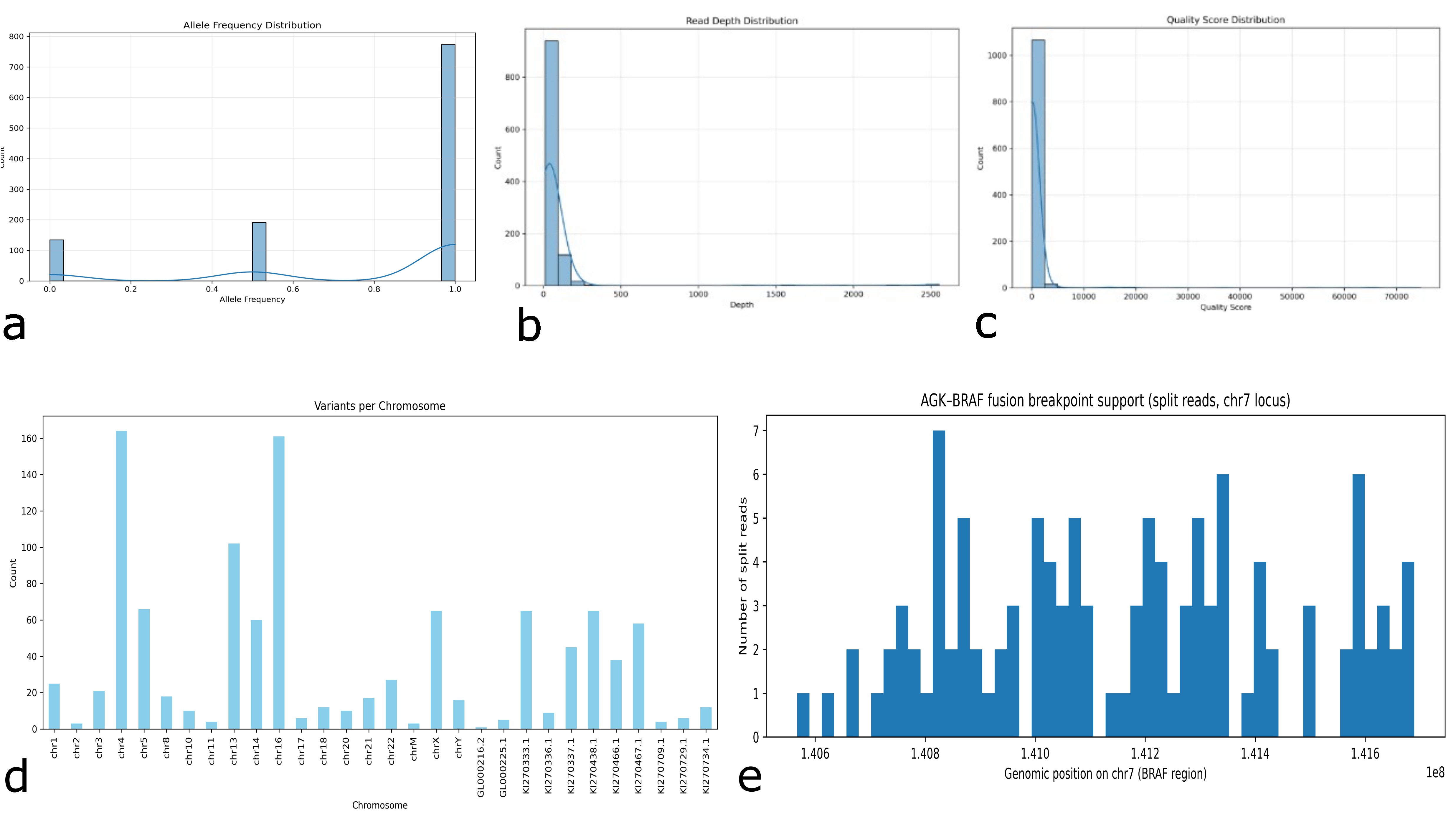

Supplement: Supplementary file 1 [file Image1.jpeg]

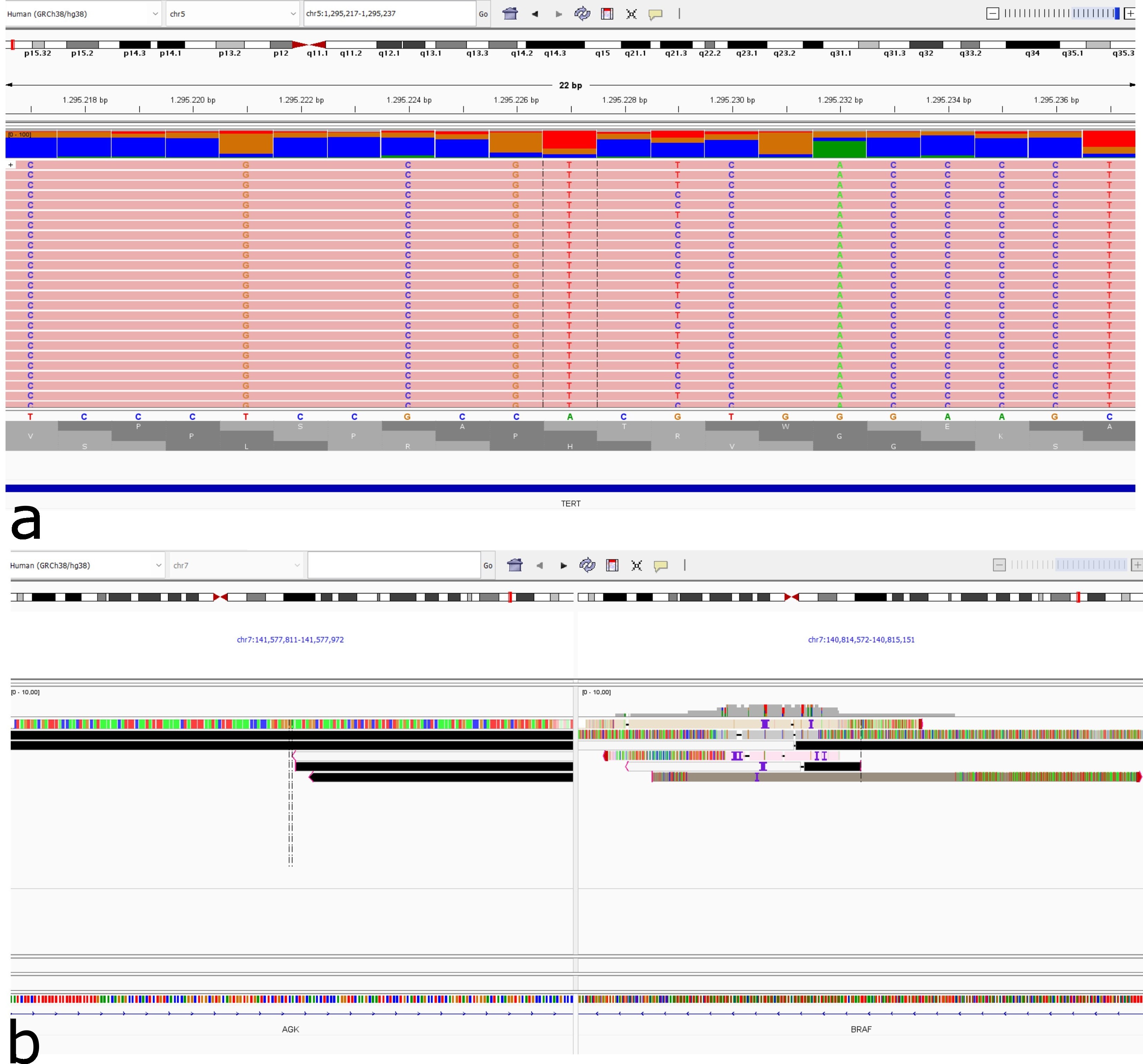

Supplement: Supplementary file 2 [file Image2.jpeg]

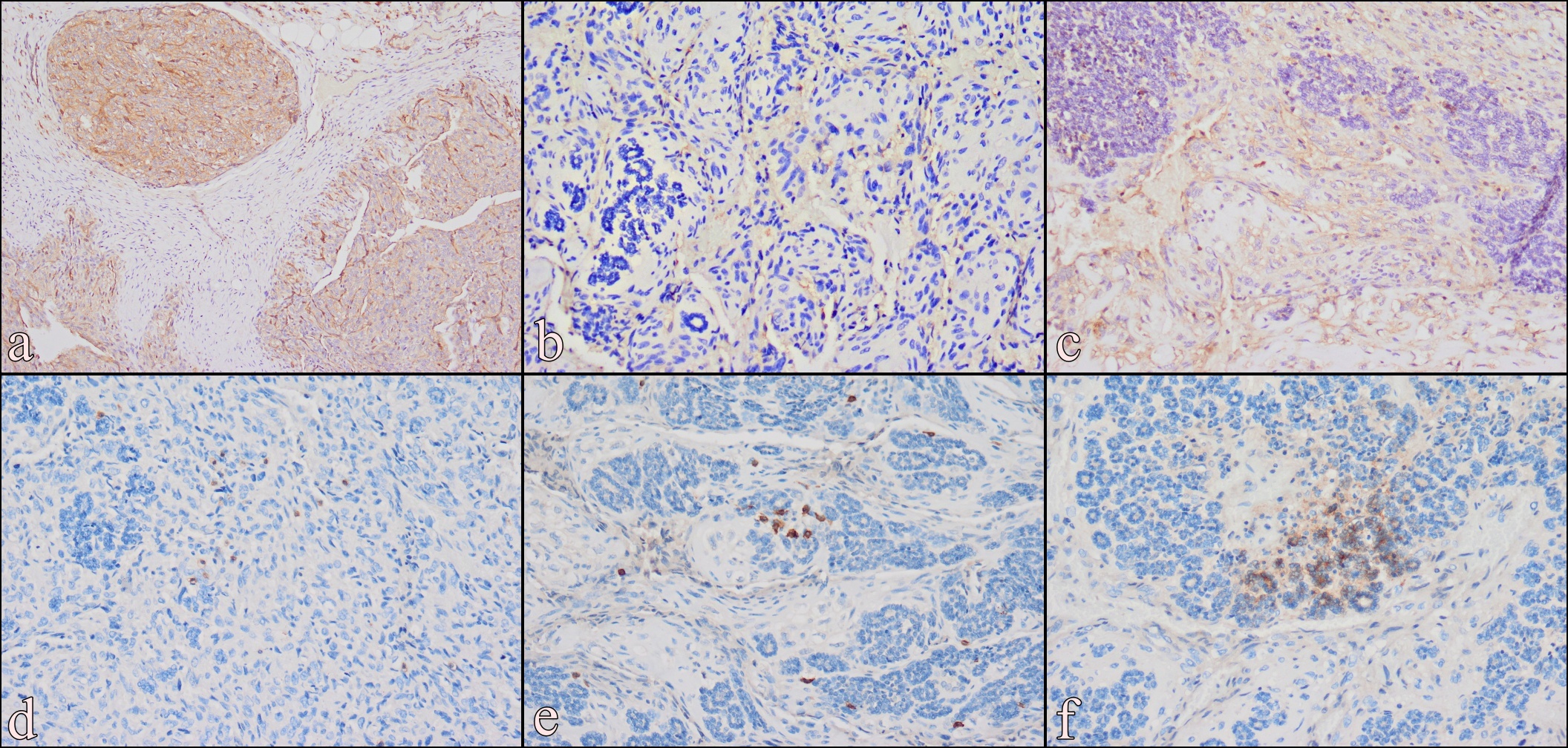

Supplement: Supplementary file 3 [file Image3.jpeg]
